# Safeguarding Female Reproductive Health Against Endocrine Disrupting Chemicals—The FREIA Project

**DOI:** 10.3390/ijms21093215

**Published:** 2020-05-01

**Authors:** Majorie B. M. van Duursen, Julie Boberg, Sofie Christiansen, Lisa Connolly, Pauliina Damdimopoulou, Panagiotis Filis, Paul A. Fowler, Bart M. Gadella, Jan Holte, Kersti Jääger, Hanna K. L. Johansson, Tianyi Li, Séverine Mazaud-Guittot, Anne-Simone Parent, Andres Salumets, Ana M. Soto, Terje Svingen, Agne Velthut-Meikas, Eva Bay Wedebye, Yuling Xie, Martin van den Berg

**Affiliations:** 1 Department Environment and Health, Vrije Universiteit Amsterdam, De Boelelaan 1085, 1081 HV Amsterdam, The Netherlands; 2 National Food Institute, Technical University of Denmark, DK-2800 Kongens Lyngby, Denmark; jubo@food.dtu.dk (J.B.); sochr@food.dtu.dk (S.C.); hakljo@food.dtu.dk (H.K.L.J.); tesv@food.dtu.dk (T.S.); ebawe@food.dtu.dk (E.B.W.); 3 The Institute for Global Food Security, School of Biological Sciences, Queen’s University Belfast, Belfast BT9 5DL, Northern Ireland, UK; l.connolly@qub.ac.uk (L.C.); YULING.XIE@qub.ac.uk (Y.X.); 4 Division of Obstetrics and Gynecology, Department of Clinical Science, Intervention and Technology, Karolinska Institutet and Karolinska University Hospital, SE-14186 Stockholm, Sweden; pauliina.damdimopoulou@ki.se (P.D.); tianyi.li.2@ki.se (T.L.); 5 Institute of Medical Sciences, School of Medicine, Medical Sciences & Nutrition, Foresterhill, Aberdeen AB23 8ZD, UK; pfilis@abdn.ac.uk (P.F.); p.a.fowler@abdn.ac.uk (P.A.F.); 6 Department of Population Health Sciences, Faculty of Veterinary Medicine, Utrecht University, Yalelaan 2, 3584 CM Utrecht, The Netherlands; b.m.gadella@uu.nl (B.M.G.); m.vandenberg@uu.nl (M.v.d.B.); 7 Carl von Linné Clinic, Uppsala Science Park, S-751 83 Uppsala, Sweden; jan.holte@linne.se; 8 Department of Obstetrics and Gynaecology, Institute of Clinical Medicine, University of Tartu and Competence Centre on Health Technologies, Teaduspargi 13, 50411 Tartu, Estonia; kerstijaager@gmail.com (K.J.); andres.salumets@ccht.ee (A.S.); 9 Univ Rennes, Inserm, EHESP, Irset (Institut de recherche en santé, environnement et travail)—UMR_S 1085, F-35000 Rennes, France; severine.mazaud@univ-rennes1.fr; 10 Neuroendocrinology Unit, GIGA-Institute, University of Liège, Belgium.1, Avenue de l’hôpital, 4000 Liège, Belgium; asparent@uliege.be; 11 Department of Immunology, Tufts University School of Medicine, Boston, MA 0211, USA; ana.soto@tufts.edu; 12 Department of Chemistry and Biotechnology, School of Science, Tallinn University of Technology, 12618 Tallinn, Estonia; agnevelthut@gmail.com

**Keywords:** female reproductive health, endocrine disrupting chemicals, test methods, risk assessment, fertility, ovary, oocyte, adrenal, mammary gland, steroidogenesis, bioassay

## Abstract

Currently available test methods are not well-suited for the identification of chemicals that disturb hormonal processes involved in female reproductive development and function. This renders women’s reproductive health at increasing risk globally, which, coupled with increasing incidence rates of reproductive disorders, is of great concern. A woman’s reproductive health is largely established during embryonic and fetal development and subsequently matures during puberty. The endocrine system influences development, maturation, and function of the female reproductive system, thereby making appropriate hormone levels imperative for correct functioning of reproductive processes. It is concerning that the effects of human-made chemicals on the endocrine system and female reproductive health are poorly addressed in regulatory chemical safety assessment, partly because adequate test methods are lacking. Our EU-funded project FREIA aims to address this need by increasing understanding of how endocrine disrupting chemicals (EDCs) can impact female reproductive health. We will use this information to provide better test methods that enable fit-for-purpose chemical regulation and then share our knowledge, promote a sustainable society, and improve the reproductive health of women globally.

## 1. Introduction

The number of women with reproductive health problems is increasing globally and scientific knowledge clearly points towards exposure to human-made chemicals being a contributing factor. It is clear that many chemicals in our food and the environment can disrupt endocrine processes and thereby threaten the reproductive health of humans, livestock, and wildlife globally [1]. These chemicals are collectively called endocrine disrupting chemicals (EDCs): “an exogenous substance or mixture that alters function(s) of the endocrine system and consequently causes adverse health effects in an intact organism, or its progeny, or (sub) populations” [2]. Numerous concerns about reproductive effects in humans and wildlife originate from findings linking exposure to EDCs in the womb to testicular dysgenesis syndrome, characterised by declining sperm counts and increasing prevalence of undescended testes, testicular cancer, and urinary duct malformation in males [3]. However, in contrast to male reproduction, we know surprisingly little about the mechanisms by which EDCs can impair female reproduction. Inevitably, therefore, the definition of ovarian dysgenesis syndrome is much more loosely encompassed [4]. This incomplete knowledge about cause–effect relationships between chemical insult and disease manifestation in women is partly the reason why we lack good test methods to address this in regulatory chemical risk assessment practice. In addition, the development of internationally recognised test methods has not kept up with the increasing scientific knowledge base. This means that women, and society more broadly, are not properly protected against potentially harmful chemicals that we encounter in our daily lives. The project FREIA (http://www.freiaproject.eu), funded under the EU Horizon 2020 programme, aims to address this pressing problem. Together with seven other projects, FREIA is part of the cluster EURION: European Cluster to Improve Identification of Endocrine Disruptors (http://www.eurion-cluster.eu).

The core objective of FREIA is to provide improved testing capabilities for the identification of EDCs that are harmful for female reproduction, as is expressed in the full project title “Female Reproductive toxicity of EDCs: a human evidence-based screening and Identification Approach”. European law requires chemicals to go through a safety evaluation before being allowed on the market. Regulators assess the endocrine disrupting properties of a chemical mostly based on data from standard test protocols that are agreed upon by the Organisation for Economic Co-operation and Development (OECD) and described in the Guidance Document on Standardised Test Guidelines for Evaluating Chemicals for Endocrine Disruption (OECD GD150) [5]. Unfortunately, currently available protocols are not well-suited for detection of EDC effects on important health outcomes, including endpoints concerning the female reproductive system. Standardised test guidelines for in vivo toxicity testing mainly cover gross, observational endpoints. These include ovarian morphology, primordial follicle counts, oestrous cyclicity, or indirect indices of female reproduction, such as pregnancy rate and pup survival. The available standardised in vitro and in silico bioassays within OECD GD150 focus mainly on effects related to (o)estrogen-androgen-thyroid-steroidogenesis (EATS) modalities. Clearly, these endpoints are too limited and do not take into full consideration the effects on the female reproductive system as a result of early life exposure to chemicals—i.e., in the womb, during infancy and puberty—when a female body is still under development. Nor do they consider lifelong exposures and (premature) reproductive senescence. In this project report, we will first briefly describe the sensitive windows during female development and how exposure to EDCs during these life stages may lead to female reproductive toxicity. Thereafter, we will highlight some gaps in current test strategies for EDC identification along with the FREIA project strategy to address this.

## 2. EDC Exposure and Female Reproductive Disorders: The Current State of Knowledge

The FREIA project focuses in particular on effects related to ovarian function, including the role of various steroid hormones and gonadotropins. The project also comprises the development of the mammary gland from the fetal stage to adulthood, because mammary gland development is known to be a sensitive endpoint for EDC identification. We acknowledge that this is only part of what constitutes ‘female reproductive health’.

### 2.1. Female Reproductive Health

Good reproductive health is important for the health and well-being of women and, if they wish to conceive, for the health of their children and future generations. Estimations show that up to one in six couples in Western countries experience problems conceiving or carrying pregnancy to term; however, the prevalence of subfertility varies widely between studies due to differences in study design and classifications [6]. A woman’s fertility decreases as she gets older, but even during her most fertile years, lifestyle choices and external factors can affect her chances of having a healthy baby. Ovulation disorders account for infertility in about 1 in 4 infertile couples. Examples of ovulation disorders are polycystic ovary syndrome (PCOS) and primary ovarian insufficiency (POI) also known as premature ovarian failure (POF). A disrupted hormone balance is a clear manifestation of these disorders and often appears to be an underlying mechanism in the aetiology. Therefore, it is not surprising that exposure to EDCs is associated with reduced female fertility and ovarian disorders. For example, epidemiological studies show that women with PCOS have higher levels of bisphenol A (BPA) [7], and perfluorooctanoic acid and perfluoro-octanesulfonate [8] compared to women without PCOS. There are strong indications to link EDC exposure to POI, however, except for cigarette smoking, there is a lack of relevant human data addressing this [9]. Furthermore, disturbances of hormone levels—e.g., by BPA [10]—can cause menstrual cycle irregularities as well as affect the quantity and quality of available oocytes.

### 2.2. Characterisation of Susceptible Windows

A woman’s reproductive health is largely established during embryonic and fetal development and later, during puberty. Female reproductive disorders in adulthood can originate from interference with hormone levels and function during development, as was clearly demonstrated by the “DES disaster”, where overt reproductive effects have been described in children born from women taking the synthetic oestrogen diethylstilbestrol (DES) as a drug during pregnancy. The adverse health effects include a rare form of vaginal cancer in girls, increased incidence of uterine fibroids, endometriosis, impaired fecundity and earlier age at menopause [11]. Startlingly, reproductive effects are still apparent in women whose mothers have been prenatally exposed to DES [12].

**Figure 1:**
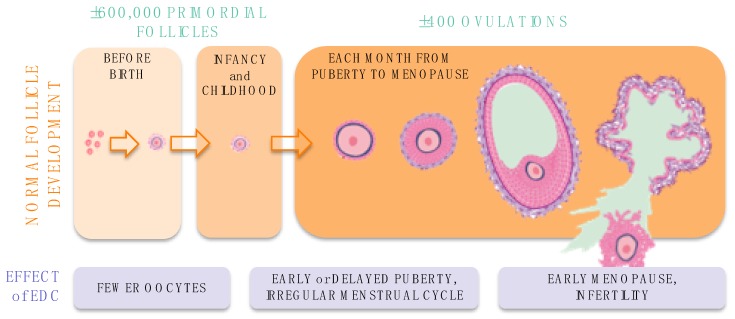
Stages of oocyte development: disruption by EDCs at different life stages leads to different reproductive effects in women. EDC, Endocrine Disrupting Chemical.

In the transition from (unborn) child to an adult woman, many hormonal processes are activated or reactivated. This means that basic biology of the female reproductive system is very different in the embryo, fetus, newborn, young girl, adolescent, and adult women. Consequently, the effect of EDC exposure on the female reproductive system depends on the age or life stage when exposure occurs (Figure 1). In addition, the effects of EDC exposure during early life may be activated or worsened by additional EDC exposure throughout a woman’s life.

### 2.3. In the Womb

At birth, a baby girl has per ovary approximately 300,000 primordial follicles containing immature eggs or oocytes, which constitutes the ovarian reserve [13]. The human ovarian reserve is finite, as was recently demonstrated by the lack of oocyte stem cells in single-cell analyses of adult human ovaries, demonstrating that lost oocytes cannot be replaced [14]. This clearly shows that the number of oocytes is a major limiting factor in human reproduction. The ovarian reserve is established through a series of critical steps during development: after migration of the primordial germ cells from the proximal epiblast to the primitive gonadal structure, the bipotential gonad differentiates into an ovary in embryos with the female genotype (XX). Next, germ cell nest formation, meiotic division (until prophase I stage), and primordial follicle assembly (involving nest breakdown, germ cell death, and formation of primordial follicles) take place, leading to the establishment of the maximal ovarian reserve at birth. The possible actions of chemicals, including EDCs such as BPA and bis(2-ethylhexyl) phthalate (DEHP), on the developing oocytes and ovary have been described in a review by Johansson et al. [15]. During pregnancy, human fetal adrenals are essential for steroid hormone production. Strikingly, the hypothalamic-pituitary-adrenal axis of the female seems more susceptible to early-life programming than that of the male [16] and thus potentially for EDCs. Several in vitro studies show interaction of EDCs with adrenal steroidogenesis, which differs from interactions with gonadal steroidogenesis [17,18]. However, how this affects female programming of the reproductive system is unknown. Undeniably, there are gaps in our scientific understanding on how disrupted endocrine and developmental processes during specific life-stages can affect ovarian and oocyte development and fertility.

### 2.4. Puberty

In girls, puberty typically starts between the age of 10–14 years, and is activated by hormonal signalling. Puberty is characterised by ovarian and adrenal maturation. The transition into puberty is driven by the reactivation of the pulsatory release of gonadotropin-releasing hormone (GnRH) by the hypothalamus leading to gonadarche—i.e., ovarian maturation—increased sex steroid production and initiation of adult pattern folliculogenesis. In humans, ovarian development in puberty is preceded by adrenarche, the maturation of the adrenal cortex, and associated increased secretion of the adrenal androgens dehydroepiandrosterone (DHEA) and its sulfate (DHEAS). This adrenarche component of puberty is unique to humans and some other higher primates. Both hormonal signalling and pubertal onset are susceptible to disturbances resulting from EDC exposure. Several studies have shown that early-life exposure to DES interferes with the programming of pulsatile GnRH secretion and feedback regulation at a time when endogenous oestradiol levels are low [19]. Likewise, changes in GnRH pulsatory release are seen upon postnatal exposure to phthalates and BPA in rodent studies [20,21,22]. This makes it highly probable that EDCs may affect pubertal onset in girls. The windows of sensitivity appear to be the prenatal and early postnatal life as well as puberty itself [23]. Globally, a trend toward earlier onset of puberty has been observed in girls. However, epidemiological studies show mixed results in relation to chemical exposures, with some exposures being associated with earlier timing of puberty in girls, and some with later [24]. Additionally, associations between EDCs and early breast development in girls has increasingly been described over the last decade [25,26]. In rats, indicators of cancer induction were observed in mammary glands after prenatal or neonatal exposure to BPA [27,28]. Clearly, EDCs can affect processes that mark the beginning of puberty. Yet, the exact processes that trigger the aforementioned effects on puberty and the influence EDCs may have thereon, still need to be clarified.

### 2.5. Adulthood

During a woman’s reproductive years (typically between 15 and 49 years of age), approximately 400 follicles will mature to the ovulatory stage until menopause marks the end of the ability to conceive a child. Each month, several primordial follicles will start to mature, but typically only a single oocyte completes the maturation process (folliculogenesis) every month. The follicles that do not complete folliculogenesis will die by atresia. During folliculogenesis, a follicle expands in size via oocyte growth, granulosa cell proliferation, theca cell recruitment from the stroma, and their subsequent proliferation. The granulosa cells synthesise oestrogens from the androgens supplied by the theca cells. Similar to the human testis, an alternative steroid producing pathway (“backdoor pathway”) is suggested based on gene and protein expression profiles [29]. However, actual levels of these backdoor steroid hormones and their roles in reproductive processes have not yet been demonstrated in the female. Moreover, it is yet to be determined how the production of hormones in the ovary specifically is affected by EDCs.

To maintain the periodical maturation and ovulation of oocytes and have a regular menstrual cycle, correct signalling of the hypothalamic-pituitary-gonadal (HPG) axis is an essential prerequisite. This ensures that the ovaries produce appropriate levels of hormones at the correct time. In mammals, including humans, the HPG axis is under negative- and positive feedback control of the sex steroids. An enigmatic feature in women is the switch from negative- to positive-feedback actions of oestradiol in the preovulatory phase, leading to a luteinizing hormone (LH) surge that triggers ovulation [30]. It is clear though, that some EDCs can affect menstrual cyclicity. For example, an in-depth review clearly shows that an alteration of the HPG axis regulation of oestrogens by BPA is an essential mechanism underlying the observed menstrual cycle irregularities observed with BPA exposure [10]. Some studies show that the rate of folliculogenesis and steroidogenesis are reduced in women treated with ketoconazole during fertility treatment [31,32]. Furthermore, a few small-scale studies indicate that EDCs, such as perfluorinated compounds and other persistent organic pollutants, in follicular fluid affect oocyte competence and artificial fertility treatment success [33]. Whether EDCs can affect growth and/or maturation of follicles or accelerate their loss has not yet been thoroughly addressed.

## 3. Advancing Test Methods for EDC Identification in Chemical Regulations

In order to achieve the main goal of FREIA and improve test methods for identification of EDCs that cause female reproductive toxicity, we need human-relevant biomarkers for ovarian toxicity spanning fetal development to childhood, puberty, and adulthood. The FREIA project has a keen focus on molecular characterisation of human ovarian and adrenal tissues and how they are affected by EDC exposure. In uniquely powered human fetal studies, ovaries and adrenals from elective terminations of normally-progressing pregnancies are being used, along with ovarian tissue donated by adult patients. In FREIA, we culture these human tissues in vitro and expose these to selected chemicals, i.e., DES (to target oestrogen receptor) and ketoconazole (to target steroidogenesis). Next, we analyse effects on morphological, protein, endocrine, and transcriptional level using a range of test methods. The focus on both human ovary and adrenal is important here, considering that the human fetal zone is not replicated in routine test animals. Moreover, steroidogenic pathways appear to be tissue-specific, as depicted above, and sex-specific. For example, in the second trimester, circulating levels of the major adrenal hormone DHEA in the female human fetus are not significantly different from serum and testicular levels the male at the same age, while intra-ovarian levels are much lower [34]. The outcomes of the human tissue studies will be extremely valuable in assessing sensitive windows of exposure and human-specific biomarkers of developmental perturbation. We also include two existing fertility treatment patient cohorts from Sweden and Estonia in the FREIA project. These cohorts enable us to assess EDC levels in follicular fluid and gene expression in granulosa cells and link these with fertility treatment outcome. Previous small-scale studies of women visiting fertility clinics have shown that human follicular fluid samples contain a wide array of chemicals with endocrine activity, such as dichlorodiphenyltrichloroethane (DDT), phthalates, BPA, and perfluorinated compounds, indicating direct exposure of maturing oocytes and their surrounding steroid-producing cells [33,35,36,37]. In addition, we carry out rodent in vivo studies, enabling us to link changes in molecular and cellular markers in the ovary, adrenal and hypothalamus during different developmental stages with actual fertility parameters in adult animals. Together, the outcomes of the human and animal studies will provide toxicity signatures for EDC-associated female reproductive toxicity. These toxicity signatures will be made publicly available in the TOXsIgN database [38] and can be used as scientific basis for the development of a strategy to address female reproductive toxicity.

### 3.1. Mechanistic Descriptions of Female Reproductive Toxicity

There is a global trend to move away from standardised animal-based testing for endocrine disrupting properties of chemicals to more human-relevant methods that are based on our understanding of mechanisms of toxicity. To date, studies reporting associations between exposure to EDCs and altered development or function of the ovaries with female reproductive toxicity are, perhaps inevitably, mostly descriptive. Thus, they provide limited information on the mechanisms driving the effects caused by EDCs. This is problematic since we need mechanistic understanding to adhere to the recently adopted criteria for identification of pesticides and biocides with endocrine disrupting properties. These criteria require information on the endocrine mode of action of a chemical, the adverse effect and a ‘biologically plausible link’ between the endocrine mode of action and the adverse effect [39]. A solution to this challenge could be to create conceptual frameworks to describe the progression of an adverse outcome, from its molecular initiating event (MIE) to secondary, tertiary, and downstream cascading key events (KEs) at the cellular, tissue, or organism level leading eventually to a pathology or adverse outcome, termed an “Adverse Outcome Pathway” (AOP). For female reproduction, this type of information is very limited. The AOPWiki, an open-source database for AOPs, only contains one description of female reproductive toxicity (https://aopwiki.org/aops/7), which is mainly based on rodent data. It pertains to reduced aromatase levels leading to reduced oestrogen synthesis, altered cyclicity, and ultimately impaired fertility. Clearly, this is severely inadequate in covering the different susceptible windows and molecular pathways that most likely will underpin EDC-related female reproductive toxicity in humans.

**Figure 2:**
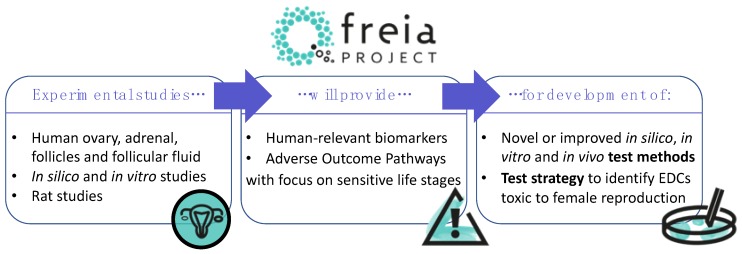
Experimental studies performed in the FREIA project will enable improved understanding of the harmful effects of endocrine disrupting chemicals (EDCs) on female reproduction. These studies will provide human-relevant biomarkers and adverse outcome pathways (AOPs) that will be used to develop dedicated test methods to identify EDCs that cause female reproductive toxicity. Furthermore, these advances will lead to a strategy around implementation of the new or improved AOPs and test methods within in regulatory chemical safety assessment.

To address this obvious shortcoming, we will start by compiling putative AOPs (pAOPs) for female reproductive toxicity with a focus on ovarian development and function (Johansson et al., submitted for publication). These pAOPs will guide our efforts towards pinpointing test methods that can best capture key events targeted by chemicals and leading up to female reproductive disorders. They will also highlight important knowledge gaps that we need to focus on in the years to come to elaborate a more robust testing strategy aimed at safeguarding women’s reproductive health. Specifically, we intend to use the newly constructed pAOPs to develop an integrated approach to testing and assessment (IATA) or ‘FREIA test strategy’ for female reproductive toxicity. Ultimately, this will enable us to prepare a guidance document that describes how to use and interpret the FREIA test strategy on female reproductive toxicity (Figure 2).

### 3.2. Dedicated Endpoints and Bioassays to Address EDC-Related Female Reproductive Toxicity

A better mechanistic understanding of the processes that direct normal ovarian development and function will help us identify the best test methods that are needed to capture key steps leading to female reproductive disorders, which is the ultimate goal of FREIA. Considering that chemical safety assessment is still largely dependent on in vivo rodent studies, it is important to characterise relevant endpoints and maximise predictivity for female reproductive toxicity in these studies. Some in vivo endpoints that FREIA addresses are listed below (this is not intended to be an exhaustive list). In addition, we will explore the potential to include these (optimised) endpoints in existing OECD test guidelines, when deemed feasible.

(1) Ovarian follicle counts performed in adult animals is included in selected OECD test guidelines, including the Extended One-Generation Reproductive Toxicity Study (TG 443). FREIA aims to improve follicle counting as currently recommended methods are highly affected by the size of the ovary and does not necessarily reflect the total follicle number.
(2) Comparison of gene expression changes in key cell types in rat ovaries and in granulosa cells from human follicular fluids will help us to find biomarkers for oocyte quality, which are currently lacking in OECD test guidelines.
(3) Morphological changes in early mammary development by image analysis of mammary gland whole mounts are considered sensitive markers of endocrine disruption in female rodents, yet is currently not part of any OECD test guideline. We will improve whole mount examination in the rat mammary gland. An unbiased and sensitive software tool will be applied for examination of mammary gland whole mounts collected at young age [40].
(4) Puberty is a time with great hormonal changes, including a dramatic increase in DHEA over the adrenarche, yet this is largely overlooked in current test strategies dealing with EDCs. In FREIA we will evaluate the need for implementation of elements of the female pubertal assay, an established protocol (US EPA OPPTS 890.145), into current or future OECD test guidelines.
(5) Changes in GnRH secretion preceding pubertal onset is considered a key event in ovarian dysfunction after EDC exposure. The effects of DES and ketoconazole on pulsatile GnRH release and hypothalamic transcriptional markers will be studied in primary rodent hypothalamic explants and compared with effects on GnRH release of in vivo exposed hypothalamus. This will allow the study of effects of prenatal exposure on the programming of maturation of GnRH secretion.

In FREIA, we use carefully designed, fit-for-purpose in silico and in vitro models with relevant cell types to experimentally assess and quantify putative molecular and cellular events underlying EDC-induced female reproductive toxicity. These models will be optimised, and standardised protocols will be provided at the end of the FREIA project. These include, but are not limited to:

(1) Quantitative structure-activity relationships (QSARs) allow us to predict molecular targets based on physico-chemical properties of a chemical. We will improve existing QSARs for aromatase and PPAR-gamma, two of the key events in a proposed adverse outcome pathway for female reproductive toxicity. These QSAR models and predictions will be made publicly available via the freely available DTU Danish (Q)SAR database (http://qsar.food.dtu.dk/) to open the possibility of integrating dedicated QSARs into toxicological risk assessment strategies.
(2) Oestrogen receptor ER-beta (ESR2) plays a central role in ovarian development and function [41], yet concentration-effect data of EDCs on this receptor is limited. A high content analysis (HCA) assay utilizing an enhanced green-fluorescence tagged ER-beta nuclear receptor transfected into the U2OS cell line will be optimised and employed for screening of selected and human relevant EDCs over a concentration range for ER-beta concentration-response activity.
(3) G-coupled protein oestrogen receptor (GPER) is suggested to act as selector during folliculogenesis [42] and may be involved in various ovarian pathologies [43]. GPER is also often more highly expressed in human fetal tissues than ER-alpha (ERS1) or ESR2. A novel high content analysis (HCA) assay for the GPER will be developed alongside HCA assays to assess subtle pre-lethal cytotoxicity in female reproductive cells.
(4) It is not known whether the standardised steroidogenic assay OECD TG 456, which uses the adrenal corticocarcinoma cell line H295R and focuses only on progesterone, testosterone, and oestradiol synthesis, is sufficiently applicable to describe (interactions with) ovarian-specific steroidogenesis. This issue will be clarified by comparing steroidomic profiles obtained from human fetal ovarian and adrenal gland cultures, adult primary human ovarian tissue cultures, and human-derived granulosa cell lines.
(5) For obvious ethical reasons, in vitro studies to assess human oocyte maturation and competency after fertilisation cannot be used for routine screening of EDCs in a regulatory context. In contrast, bovine ovarian tissues and follicles can easily be obtained from slaughterhouses. From these collected follicles, oocytes can be isolated and matured and used for in vitro fertilisation experiments, where embryonic development can be followed routinely until blastocyst hatching and elongation. Several studies have demonstrated the value of such bovine oocytes as experimental model for human reproductive parameters [44]. This test method adheres fully to the 3R-principle of toxicity testing in the 21st Century. Bovine oocyte studies will be performed to assess the effects of EDCs on oocyte maturation and the post-fertilisation competence to develop into a healthy embryo will be explored.

### 3.3. Testing for EDCs in the Regulatory Context

In the European Union, chemicals have to go through a safety evaluation before being allowed on the market. The type of information that needs to be provided by industry to the regulators, however, depends as much on the production volume and intended application for the chemical as on its inherent properties. In other words, the required toxicity tests depend on whether a chemical is a pesticide, biocide, food additive, cosmetics additive, or an industrial chemical. Specific criteria and guidance for identification of EDCs only exist for pesticides and biocides [39]. In the REACH regulation, endocrine disrupting properties of industrial chemicals are currently assessed case by case for substances of very high concern and is based on existing scientific evidence and expert opinion. Other regulations addressing the safety of chemicals in everyday products—such as cosmetics, toys, or food contact materials—currently do not have specific identification processes for EDCs. Obviously, there is still much to gain in regulatory procedures and risk management strategies for EDCs, and improved test capabilities will certainly contribute to better protection of health and the environment [45]. As described above, FREIA aims to improve capabilities for EDC identification in chemical regulations by providing improved or additional endpoints in existing OECD test guidelines and provide new assays. We work closely with seven other EU-funded projects in a cluster called EURION. The other projects in the EURION cluster develop test methods to identify EDCs that cause thyroid hormone disruption, developmental neurotoxic effects, and/or metabolic diseases. Together, these projects will provide robust test methods that have the potential to improve EDC identification in various regulatory chemical frameworks. Internationally, we are faced with the added problem that chemical regulation and legislation is different in different parts of the world. Here, the International Advisory Panel of the EURION cluster may play a role by providing a bridge to other European and international initiatives and regulatory bodies. The FREIA consortium is a proponent of a more harmonised regulatory framework that puts people’s health at the forefront, irrespective of where in the world the individual happens to reside or what application a specific chemical was intended for.

## 4. Sharing and Safeguarding

Clearly, the outcomes of the FREIA project will significantly improve our understanding of female reproductive toxicity and, more specifically, how EDCs contribute. Together with outcomes of the European Human Biomonitoring Initiative (HBM4EU) and the Scottish Advanced Fetal Research (SAFeR) Study, we can make important contributions to our understanding of how lifestyle factors can influence EDC-related disease risk, specifically regarding female reproductive health. While EDC exposures during early development might prime a woman’s predisposition to compromised reproductive health, these effects can be amplified through additional exposures or lifestyle factors after birth. After all, the ovarian reserve should last for decades, yet, it is continuously exposed to EDCs. The scientific knowledge generated in the FREIA project will provide openings to map out options to improve female reproductive health, for example through lifestyle choices that may reduce exposure to EDCs. These options will be discussed with relevant stakeholders and gathered in a health promotion strategy that builds on existing expertise (e.g., from the WHO, EFSA, EPA) and (grass root) initiatives, such as “toxin-free Stockholm” (miljobarometern.stockholm.se/miljogifter/kemikaliecentrum/). We have established partnerships with major actors in policy and advocacy on (women’s) health protection. These include the Health and Environment Alliance (HEAL), the International Federation of Gynecologists and Obstetrics (FIGO), and the International Federation of Fertility Societies (IFFS). These partnerships allow us to communicate our scientific findings in light of the importance of a healthy lifestyle and a sustainable environment, and to inform society how to avoid the potential health risks associated with EDCs.

## 5. Conclusions

The main objective of the FREIA project is to provide dedicated, human-relevant, test methods to identify EDCs that cause female reproductive toxicity, before the end of 2023. The FREIA consortium consists of 11 partners with outstanding scientific and regulatory expertise on endocrine disruption in relation to women’s reproductive health, early life development, epidemiology, endocrinology, and toxicology. Our combined expertise, the availability of human female tissues, together with advances in international AOP frameworks, provide an unparalleled opportunity to improve existing test methods and develop dedicated novel in vitro and in silico methods to identify EDCs that affect female reproductive health. The FREIA project has a strong focus on developing test methods and strategies that have the widest reach and at the same time are harmonised enough to allow inclusion in internationally validated and accepted guidelines, such as the OECD TG conceptual framework for testing and assessment of endocrine disruptors [5]. Moreover, the FREIA project aims to identify and close some knowledge gaps by providing increased understanding of female reproductive toxicity, which can be used by scientists and other stakeholders beyond the scientific community to safeguard women’s reproductive health against EDCs. Obviously, the FREIA project will not solve the entire problem of EDC-induced female reproductive disorders, but it is our hope, and intention, to at least move the guidepost far enough that the next generation of women is better protected against EDCs than the present generation.

## Abbreviations

AOP	Adverse outcome pathway
BPA	Bisphenol A
DDT	Dichlorodiphenyltrichloroethane
DEHP	Bis(2-ethylhexyl) phthalate
DES	Diethylstilbestrol
DHEA(S)	Dehydroepiandrosterone(-sulfate)
EATS	Estrogen-androgen-thyroid-steroidogenesis
EDC	Endocrine disrupting chemical
FREIA	“Female Reproductive toxicity of EDCs: a human evidence-based screening and Identification Approach”
FSH	Follicle stimulating hormone
GnRH	Gonadotropin-releasing hormone
GPER	G-coupled protein oestrogen receptor
HP(A)G axis	Hypothalamic-pituitary-(adrenal)-gonadal axis
IATA	Integrated approach to testing and assessment
LH	Luteinizing hormone
OECD	Organisation for Economic Cooperation and Development
PCOS	Polycystic ovary syndrome
POF	Premature ovarian failure
POI	Primary ovarian insufficiency
REACH	Registration, Evaluation, Authorisation and restriction of CHemicals
TG	Test guideline

## References

[1] WHO/UNEP (2013). State of the Science of Endocrine Disrupting Chemicals—2012.

[2] WHO/IPCS (2002). Global Assessment of the State-of-the-Science of Endocrine Disruptors.

[3] Toppari J., Virtanen H.E., Main K.M., Skakkebaek N.E. (2010). Cryptorchidism and hypospadias as a sign of testicular dysgenesis syndrome (TDS): Environmental connection. Birth Defects Res. A Clin. Mol. Teratol..

[4] Buck Louis G., Cooney M., Peterson C. (2011). The ovarian dysgenesis syndrome. J. Dev. Orig. Health Dis..

[5] OECD Revised Guidance Document 150 on Standardised Test Guidelines for Evaluating Chemicals for Endocrine Disruption.

[6] Farquhar C.M., Bhattacharya S., Repping S., Mastenbroek S., Kamath M.S., Marjoribanks J., Boivin J. (2019). Female subfertility. Nat. Rev. Dis Primers.

[7] Hu Y., Wen S., Yuan D., Peng L., Zeng R., Yang Z., Liu Q., Xu L., Kang D. (2018). The association between the environmental endocrine disruptor bisphenol A and polycystic ovary syndrome: A systematic review and meta-analysis. Gynecol. Endocrinol..

[8] Vagi S.J., Azziz-Baumgartner E., Sjodin A., Calafat A.M., Dumesic D., Gonzalez L., Kato K., Silva M.J., Ye X., Azziz R. (2014). Exploring the potential association between brominated diphenyl ethers, polychlorinated biphenyls, organochlorine pesticides, perfluorinated compounds, phthalates, and bisphenol A in polycystic ovary syndrome: A case-control study. BMC Endocr. Disord..

[9] Vabre P., Gatimel N., Moreau J., Gayrard V., Picard-Hagen N., Parinaud J., Leandri R.D. (2017). Environmental pollutants, a possible etiology for premature ovarian insufficiency: A narrative review of animal and human data. Environ. Health.

[10] Viguie C., Mhaouty-Kodja S., Habert R., Chevrier C., Michel C., Pasquier E. (2018). Evidence-based adverse outcome pathway approach for the identification of BPA as en endocrine disruptor in relation to its effect on the estrous cycle. Mol. Cell Endocrinol..

[11] Conlon J.L. (2017). Diethylstilbestrol: Potential health risks for women exposed in utero and their offspring. JAAPA.

[12] Titus L., Hatch E.E., Drake K.M., Parker S.E., Hyer M., Palmer J.R., Strohsnitter W.C., Adam E., Herbst A.L., Huo D. (2019). Reproductive and hormone-related outcomes in women whose mothers were exposed in utero to diethylstilbestrol (DES): A report from the US National Cancer Institute DES Third Generation Study. Reprod. Toxicol..

[13] Wallace W.H., Kelsey T.W. (2010). Human ovarian reserve from conception to the menopause. PLoS ONE.

[14] Wagner M., Yoshihara M., Douagi I., Damdimopoulos A., Panula S., Petropoulos S., Lu H., Pettersson K., Palm K., Katayama S. (2020). Single-cell analysis of human ovarian cortex identifies distinct cell populations but no oogonial stem cells. Nat. Commun..

[15] Johansson H.K.L., Svingen T., Fowler P.A., Vinggaard A.M., Boberg J. (2017). Environmental influences on ovarian dysgenesis—Developmental windows sensitive to chemical exposures. Nat. Rev. Endocrinol..

[16] Carpenter T., Grecian S.M., Reynolds R.M. (2017). Sex differences in early-life programming of the hypothalamic-pituitary-adrenal axis in humans suggest increased vulnerability in females: A systematic review. J. Dev. Orig. Health Dis..

[17] Pinto C.L., Markey K., Dix D., Browne P. (2018). Identification of candidate reference chemicals for in vitro steroidogenesis assays. Toxicol. Vitr..

[18] Roelofs M.J., Piersma A.H., van den Berg M., van Duursen M.B. (2013). The relevance of chemical interactions with CYP17 enzyme activity: Assessment using a novel in vitro assay. Toxicol. Appl. Pharm..

[19] Franssen D., Ioannou Y.S., Alvarez-real A., Gerard A., Mueller J.K., Heger S., Bourguignon J.P., Parent A.S. (2014). Pubertal timing after neonatal diethylstilbestrol exposure in female rats: Neuroendocrine vs peripheral effects and additive role of prenatal food restriction. Reprod. Toxicol..

[20] Franssen D., Gerard A., Hennuy B., Donneau A.F., Bourguignon J.P., Parent A.S. (2016). Delayed Neuroendocrine Sexual Maturation in Female Rats After a Very Low Dose of Bisphenol A Through Altered GABAergic Neurotransmission and Opposing Effects of a High Dose. Endocrinology.

[21] Kasper-Sonnenberg M., Wittsiepe J., Wald K., Koch H.M., Wilhelm M. (2017). Pre-pubertal exposure with phthalates and bisphenol A and pubertal development. PLoS ONE.

[22] Rasier G., Parent A.S., Gerard A., Lebrethon M.C., Bourguignon J.P. (2007). Early maturation of gonadotropin-releasing hormone secretion and sexual precocity after exposure of infant female rats to estradiol or dichlorodiphenyltrichloroethane. Biol. Reprod..

[23] Parent A.S., Franssen D., Fudvoye J., Gerard A., Bourguignon J.P. (2015). Developmental variations in environmental influences including endocrine disruptors on pubertal timing and neuroendocrine control: Revision of human observations and mechanistic insight from rodents. Front. Neuroendocr..

[24] Greenspan L.C., Lee M.M. (2018). Endocrine disrupters and pubertal timing. Curr Opin Endocrinol Diabetes Obes.

[25] Binder A.M., Corvalan C., Pereira A., Calafat A.M., Ye X., Shepherd J., Michels K.B. (2018). Prepubertal and Pubertal Endocrine-Disrupting Chemical Exposure and Breast Density among Chilean Adolescents. Cancer Epidemiol. Biomark. Prev..

[26] Wolff M.S., Teitelbaum S.L., McGovern K., Pinney S.M., Windham G.C., Galvez M., Pajak A., Rybak M., Calafat A.M., Kushi L.H. (2015). Environmental phenols and pubertal development in girls. Environ. Int..

[27] Acevedo N., Davis B., Schaeberle C.M., Sonnenschein C., Soto A.M. (2013). Perinatally administered bisphenol a as a potential mammary gland carcinogen in rats. Environ. Health Perspect..

[28] Mandrup K., Boberg J., Isling L.K., Christiansen S., Hass U. (2016). Low-dose effects of bisphenol A on mammary gland development in rats. Andrology.

[29] Marti N., Galvan J.A., Pandey A.V., Trippel M., Tapia C., Muller M., Perren A., Fluck C.E. (2017). Genes and proteins of the alternative steroid backdoor pathway for dihydrotestosterone synthesis are expressed in the human ovary and seem enhanced in the polycystic ovary syndrome. Mol. Cell Endocrinol..

[30] Moenter S.M., Silveira M.A., Wang L., Adams C. (2020). Central aspects of systemic oestradiol negative- and positive-feedback on the reproductive neuroendocrine system. J. Neuroendocr..

[31] Gal M., Orly J. (2014). Ketoconazole inhibits ovulation as a result of arrest of follicular steroidogenesis in the rat ovary. Clin. Med. Insights Reprod. Health.

[32] Parsanezhad M.E., Alborzi S., Pakniat M., Schmidt E.H. (2003). A double-blind, randomized, placebo-controlled study to assess the efficacy of ketoconazole for reducing the risk of ovarian hyperstimulation syndrome. Fertil. Steril..

[33] Bjorvang R.D., Damdimopoulou P. (2020). Persistent environmental endocrine-disrupting chemicals in ovarian follicular fluid and in vitro fertilization treatment outcome in women. Upsala J. Med. Sci..

[34] O’Shaughnessy P.J., Antignac J.P., Le Bizec B., Morvan M.L., Svechnikov K., Soder O., Savchuk I., Monteiro A., Soffientini U., Johnston Z.C. (2019). Alternative (backdoor) androgen production and masculinization in the human fetus. PLoS Biol..

[35] Craig Z.R., Wang W., Flaws J.A. (2011). Endocrine-disrupting chemicals in ovarian function: Effects on steroidogenesis, metabolism and nuclear receptor signaling. Reproduction.

[36] Petro E.M., Leroy J.L., Covaci A., Fransen E., De Neubourg D., Dirtu A.C., De Pauw I., Bols P.E. (2012). Endocrine-disrupting chemicals in human follicular fluid impair in vitro oocyte developmental competence. Hum. Reprod.

[37] Petro E.M.L., D’Hollander W., Covaci A., Bervoets L., Fransen E., De Neubourg D., De Pauw I., Leroy J., Jorssen E.P.A., Bols P.E.J. (2014). Perfluoroalkyl acid contamination of follicular fluid and its consequence for in vitro oocyte developmental competence. Sci. Total Environ..

[38] Darde T.A., Gaudriault P., Beranger R., Lancien C., Caillarec-Joly A., Sallou O., Bonvallot N., Chevrier C., Mazaud-Guittot S., Jegou B. (2018). TOXsIgN: A cross-species repository for toxicogenomic signatures. Bioinformatics.

[39] EFSA/ECHA (2018). Guidance for the identification of endocrine disruptors in the context of Regulations (EU) No 528/2012 and (EC) No 1107/2009. EFSA J..

[40] Montévil A.R., Acevedo N., Schaeberle C., Bharadwai M., Fenton S.E., Soto A.M. (2020). A combined morphometric and statistical approach to assess non-monotonicity in the developing mammary gland of rats in the CLARITY-BPA study. Environ. Health Perspect..

[41] Drummond A.E., Fuller P.J. (2010). The importance of ERbeta signalling in the ovary. J. Endocrinol..

[42] Heublein S., Lenhard M., Vrekoussis T., Schoepfer J., Kuhn C., Friese K., Makrigiannakis A., Mayr D., Jeschke U. (2012). The G-protein-coupled estrogen receptor (GPER) is expressed in normal human ovaries and is upregulated in ovarian endometriosis and pelvic inflammatory disease involving the ovary. Reprod. Sci..

[43] Tang Z.R., Zhang R., Lian Z.X., Deng S.L., Yu K. (2019). Estrogen-Receptor Expression and Function in Female Reproductive Disease. Cells.

[44] Santos R.R., Schoevers E.J., Roelen B.A. (2014). Usefulness of bovine and porcine IVM/IVF models for reproductive toxicology. Reprod. Biol. Endocrinol..

[45] Demeneix B., Slama R. (2019). Endocrine Disruptors: From Scientific Evidence to Human Health Protection. http://www.europarl.europa.eu/supporting-analyses.

